# Investigation of the cardiac impacts of endothelial PAS domain-containing protein 1 and ghrelin in patients with systemic lupus erythematosus

**DOI:** 10.1590/1806-9282.20241412

**Published:** 2025-03-31

**Authors:** Yakup Alsancak, Ahmet Taha Sahin, Oznur Keskin, Selman Parlak

**Affiliations:** 1 Necmettin Erbakan University, Faculty of Meram Medicine, Department of Cardiology – Konya, Turkey.; 2 Beyhekim Training and Research Hospital, Department of Cardiology – Konya, Turkey.; 3 Beyhekim Training and Research Hospital, Department of Rheumatology – Konya, Turkey.

**Keywords:** Systemic lupus erythematosus, EPAS1, Ghrelin, Right ventricular dysfunction, Pulmonary hypertension

## Abstract

**OBJECTIVE:**

Systemic lupus erythematosus is a chronic autoimmune disease with significant cardiac manifestations, including right ventricular dysfunction and pulmonary hypertension. This study aims to explore the relationship between endothelial PAS domain-containing protein 1 and ghrelin levels with right ventricular function in patients with systemic lupus erythematosus and assess their potential as biomarkers for cardiac involvement.

**METHODS:**

A prospective study was conducted involving 34 patients with systemic lupus erythematosus and 35 healthy controls. Echocardiographic parameters were recorded. Serum levels of endothelial PAS domain-containing protein 1 and ghrelin were measured using enzyme-linked immunosorbent assay. Statistical analyses included comparisons between groups and correlations between biomarkers and echocardiographic parameters.

**RESULTS:**

Patients with systemic lupus erythematosus had significantly higher levels of endothelial PAS domain-containing protein 1 and ghrelin compared with healthy controls (p<0.001). Endothelial PAS domain-containing protein 1 showed a moderate positive correlation with right ventricular systolic motion (p=0.007). Patients with systemic lupus erythematosus and a higher disease activity had elevated endothelial PAS domain-containing protein 1 levels, particularly those with positive antiphospholipid antibodies (p<0.001). No significant correlation was found between ghrelin levels and right ventricular function. Receiver operating characteristic curve analysis identified cutoff values for endothelial PAS domain-containing protein 1 (≥1.871) and ghrelin (≥360.50) with moderate sensitivity and specificity for predicting systemic lupus erythematosus.

**CONCLUSION:**

This study suggests that endothelial PAS domain-containing protein 1 and ghrelin may play important roles in the pathophysiology of right ventricular dysfunction in systemic lupus erythematosus.

## INTRODUCTION

Systemic lupus erythematosus (SLE) is a chronic autoimmune disease characterized by a complex interplay of genetic, environmental, and hormonal factors leading to the production of autoantibodies and widespread inflammation^1^. The disease mechanism primarily involves immune complex deposition, which can affect multiple organs, including the heart^2^. SLE is known to have significant cardiac implications, including myocarditis, pericarditis, and an increased risk of coronary artery disease^3^. One of the critical cardiac manifestations in patients with SLE is the involvement of right ventricular function, which can be compromised due to both direct myocardial involvement and secondary effects such as pulmonary hypertension^4,5^. Pulmonary hypertension in SLE can result from various factors, including chronic inflammation, vasculitis, and thromboembolic events, leading to right ventricular dysfunction and heart failure^6^.

Endothelial PAS domain-containing protein 1 (EPAS-1), also known as hypoxia-inducible factor 2-alpha, is a transcription factor that plays a crucial role in the cellular response to hypoxia^7^. EPAS-1 is involved in the regulation of genes associated with angiogenesis, erythropoiesis, and metabolism, which are vital for maintaining oxygen homeostasis in hypoxic conditions^8^. In the context of cardiac function, EPAS-1 has been implicated in the adaptive responses of the heart to hypoxia, including promoting angiogenesis and modulating myocardial metabolism^9^. These effects suggest that EPAS-1 may have a protective role in conditions where cardiac tissue is subjected to hypoxic stress, such as in pulmonary hypertension or right ventricular dysfunction associated with SLE.

Ghrelin, a peptide hormone primarily produced in the stomach, has been recognized for its multifaceted role in cardiovascular physiology^10^. Beyond its well-known effects on appetite regulation, ghrelin has been shown to have cardioprotective properties, including anti-inflammatory, antiapoptotic, and vasodilatory effects^11^. In heart failure, ghrelin has been observed to improve cardiac output and reduce systemic vascular resistance, making it a molecule of interest in the study of cardiovascular diseases^12^. Given its potential to modulate cardiac function, ghrelin's role in the context of SLE-related cardiac dysfunction and pulmonary hypertension warrants further investigation.

The present study aims to explore the relationship between EPAS-1 and ghrelin levels with right ventricular function and pulmonary hypertension in patients with SLE. By comparing these parameters between patients with SLE and healthy controls, this study seeks to elucidate the potential roles of EPAS-1 and ghrelin in the cardiac manifestations of SLE and their utility as biomarkers for disease severity and cardiac involvement.

## METHODS

This prospective study was conducted between January 2023 and January 2024, including 34 patients diagnosed with SLE and 35 healthy controls. Inclusion criteria for the study consisted of patients aged 18–65 years with a confirmed diagnosis of SLE according to the American College of Rheumatology criteria, who were under regular follow-up and had no history of other significant autoimmune or chronic inflammatory diseases. Exclusion criteria included patients with a history of cardiovascular diseases unrelated to SLE, such as coronary artery disease, valvular heart disease, or cardiomyopathy; chronic renal insufficiency (e.g., chronic kidney disease stage 3 or higher); or those who had undergone recent major surgery within the last 6 months. Additionally, individuals with other autoimmune or chronic inflammatory diseases, including rheumatoid arthritis, Sjögren's syndrome, or inflammatory bowel disease, were excluded. Metabolic conditions such as diabetes mellitus, uncontrolled hypertension, or dyslipidemia were also considered exclusionary. Pregnant or lactating individuals, as well as those with a history of malignancy or active cancer, were excluded to minimize confounding variables. Finally, individuals on medications or treatments known to significantly impact cardiac or vascular function independent of SLE were not included in the study.

Echocardiographic parameters were meticulously recorded for all participants. Key parameters assessed included left ventricular ejection fraction (EF), tricuspid annular plane systolic excursion (TAPSE), pulmonary pulse transit time (pPTT), and right ventricular systolic pressure. Echocardiography was performed using a standardized protocol, and measurements were taken by experienced cardiologists blinded to the study groups. Additionally, patient medication history, including the use of plaquenil, prednisolone, and mycophenolate mofetil, was documented. The SLE Disease Activity Index (SLE-DAI) score was calculated for each patient, incorporating clinical and laboratory data to assess disease activity.

Laboratory evaluations included the measurement of autoantibodies such as anti-dsDNA, anti-Smith (SM), anti-ribonucleoprotein (RNP), and extractable nuclear antigens (ENA). The presence of antiphospholipid antibodies (APLA) was also recorded. Blood samples were collected after an overnight fast, and serum levels of EPAS-1 and ghrelin were measured using enzyme-linked immunosorbent assay kits. All laboratory assessments were conducted in a central laboratory to ensure consistency and reliability of the results.

### Statistical analysis

The data obtained from the study were analyzed using the Statistical Package for Social Sciences version 25.0. Descriptive statistics were presented as frequency (n) and percentage (%) for categorical variables and as median (interquartile range, IQR) for continuous variables. The chi-square (χ^2^) test and Fisher's exact test were employed for the comparison of categorical variables. The normality of the distribution of continuous variables was assessed using the Shapiro-Wilk test. For comparisons between two independent groups with non-normally distributed continuous variables, the Mann-Whitney U test was utilized. The relationship between two non-normally distributed continuous variables was evaluated using Spearman's correlation analysis. The strength of the correlations was interpreted as follows: r=0.05–0.30 was considered a weak correlation, r=0.30–0.40 a low-moderate correlation, r=0.40–0.60 a moderate correlation, r=0.60–0.70 a good correlation, r=0.70–0.75 a very good correlation, and r=0.75–1.00 an excellent correlation. Results were considered statistically significant at a 95% confidence interval, with a significance level set at p<0.05.

## RESULTS

This study included 34 patients diagnosed with SLE and 35 healthy controls. Among the patients with SLE, 94.1% (n=34) were female, with an age distribution of 34 years (IQR: 25–43 years). The distribution of gender and age between the patient and control groups was found to be similar (p>0.05). The distribution of echocardiographic parameters between the patient and control groups is presented in Table 1. EF levels were significantly higher in patients with SLE compared with the control group, while TAPSE and pPTT levels were significantly lower (p=0.005; p=0.010; p=0.045; p<0.001). The SLE-DAI score ranged from 8 (IQR: 4–20). APLA was positive in 23.5% of the patients. Antinuclear antibodies (ANA) were positive in 73.5%, anti-dsDNA in 55.9%, and anti-Smith/RNP in 29.4% of the patients. Regarding treatment, 79.4% of patients with SLE were on plaquenil, 67.6% on prednisolone, and 35.3% on mycophenolate mofetil.

**Table 1:** Distribution of echocardiographic and laboratory data in Systemic lupus erythematosus and control groups.

	Control (n=35)	Patients with SLE (n=34)	p-value
EF (%)[median (IQR)]	60 (60–60)	62 (59–64)	**0.005** *
LVEDD (mm) [median (IQR)]	43.60 (40–49)	43 (40–48)	0.786*
LVESD (mm) [median (IQR)]	26.2 (21–30)	25 (20–29)	0.534*
IVS [median (IQR)]	9 (8–10)	9 (8–10)	0.956*
PW [median (IQR)]	9 (8.50–10)	9 (8.75–10)	0.885*
LA (mm) [median (IQR)]	29 (23–33)	28 (24–31.25)	0.661*
AO [median (IQR)]	21.50 (20–24.50)	22 (20.75–24.25)	0.723*
E (cm/s) [median (IQR)]	75 (63–86)	76.50 (63.75–86.25)	0.478*
A (cm/s) [median (IQR)]	58 (53.50–71)	62 (56.50–73.25)	0.251*
sPAP (mmHg) [median (IQR)]	25 (22–16)	26 (22.75–30.25)	0.127*
RV-SM [median (IQR)]	14.4 (13.1–16.0)	13.5 (12.0–15.1)	0.061*
TAPSE [median (IQR)]	2.5 (2.2–2.6)	2.3 (2.0–2.5)	**0.045** *
pPTT [median (IQR)]	127 (117–192)	110 (91–117)	**<0.001** *
EPAS-1 (ng/mL) [median (IQR)]	1.70 (1.49–2.09)	2.01 (1.71–3.53)	**0.007** **
Ghrelin (pg/mL) [median (IQR)]	311.00 (294.00–357.00)	393.50 (364.00–430.25)	**<0.001** **

* Fisher's exact test.

** Mann-Whitney U test. SLE: Systemic lupus erythematosus; EF: ejection fraction; IQR: interquartile range; RV-SM: right ventricular systolic motion; TAPSE: tricuspid annular plane systolic excursion; pPTT: pulmonary pulse transit time; EPAS-1: endothelial PAS domain-containing protein 1. Bold text in the tables indicates statistical significance below p<0.05.

**Figure 1:**
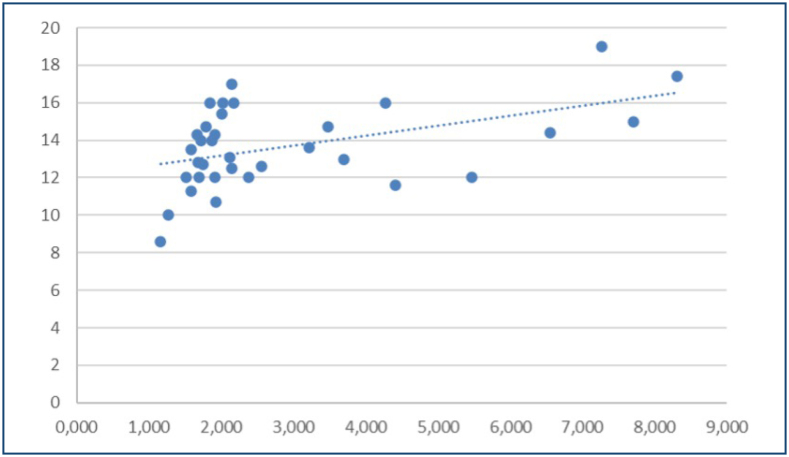
Scatter plot showing the moderate positive correlation between right ventricular systolic motion (RV-SM) and EPAS-1 levels in patients with systemic lupus erythematosus (SLE).

The distribution of EPAS-1 and ghrelin levels between the patient and control groups is presented in Table 2. EPAS-1 and ghrelin levels were significantly higher in patients with SLE compared with the controls (p=0.007; p<0.001). The correlation between ECHO right ventricular function and EPAS-1 and ghrelin levels is shown in Table 3. There was a moderate positive correlation between right ventricular systolic motion (RV-SM) and EPAS-1 (Figure 1). Patients with SLE were grouped according to SLE-DAI scores of <15 and ≥15. The distribution of EPAS-1 and ghrelin levels according to SLE-DAI groups and APLA positivity is shown in Table 3. EPAS-1 levels were significantly lower in the group with an SLE-DAI score <15 (p<0.001). EPAS-1 levels were significantly higher in the APLA-positive group compared with the negative group (p<0.001). No significant difference was found in the distribution of ghrelin levels (p>0.05).

**Table 2:** Correlation of endothelial PAS domain-containing protein 1 and ghrelin values with right ventricular functions.

		EPAS-1	Ghrelin
sPAP (mmHg)	R	-0.004	-0.008
	p	0.984	0.966
RV-SM	R	**0.449**	0.150
	P	**0.008**	0.398
TAPSE	r	0.321	0.054
	P	0.064	0.763
pPTT	r	0.054	-0.0210
	p	0.761	0.232

3 r: Spearman's correlation coefficient. EPAS-1: endothelial PAS domain-containing protein 1; RV-SM: right ventricular systolic motion; TAPSE: tricuspid annular plane systolic excursion; pPTT: pulmonary pulse transit time. Bold text in the tables indicates statistical significance below p<0.05.

**Table 3:** Distribution of disease characteristics in patients with Systemic lupus erythematosus.

		Patients with SLE (n=34)			
		EPAS-1	p-value	Ghrelin	p-value
SLE-DAI score [n (%)]					
	≥15	5.46 (3.13–7.48)	**<0.001** *	379.00 (344.00–413.50)	0.335*
	<15	1.90 (1.67–2.15)		394.00 (370.00–434.00)	
APLA [n (%)]					
	Negative	1.89 (1.67–2.14)	**<0.001** *	394.50 (377.75–432.50)	0.130*
	Positive	6.00 (4.30–7.59)		351.50 (355.75–411.00)	

* Mann-Whitney U test. SLE: Systemic lupus erythematosus; EPAS-1: endothelial PAS domain-containing protein 1; SLE-DAI: systemic lupus erythematosus-Disease Activity Index; APLA: antiphospholipid antibodies. Bold text in the tables indicates statistical significance below p<0.05.

## DISCUSSION

The findings of this study highlight the significant role of EPAS-1 and ghrelin in the pathophysiology of right ventricular dysfunction in patients with SLE. EPAS-1, a critical regulator of hypoxia-inducible responses, was found to be elevated in patients with SLE, particularly those with higher disease activity and APLA positivity. These results align with previous studies that have demonstrated the upregulation of hypoxia-inducible factors in autoimmune diseases, suggesting that EPAS-1 may serve as a marker of hypoxic stress and inflammation in SLE^13,14^.

Ghrelin, a hormone known for its cardioprotective effects, was also significantly elevated in the SLE cohort compared with healthy controls. This is consistent with studies that have shown increased ghrelin levels in conditions associated with chronic inflammation and cardiovascular stress, such as heart failure and pulmonary hypertension^15^. The lack of significant correlation between ghrelin levels and SLE disease activity in this study suggests that while ghrelin may be elevated as a compensatory mechanism, it may not directly reflect the severity of SLE-related cardiac involvement.

The observed correlation between EPAS-1 levels and right ventricular function is of particular interest. Previous research has highlighted the detrimental effects of chronic hypoxia on right ventricular function, particularly in the setting of pulmonary hypertension^16^. The moderate positive correlation between EPAS-1 and RV-SM in this study suggests that EPAS-1 may play a role in the adaptive response of the right ventricle to hypoxic stress, possibly through the promotion of angiogenesis and metabolic adaptations.

In contrast, the lack of a significant correlation between ghrelin levels and right ventricular function might indicate that ghrelin's role in SLE-related cardiac dysfunction is more complex and could involve pathways beyond direct cardiac protection. The anti-inflammatory and vasodilatory properties of ghrelin, as reported in other studies, may contribute to its cardioprotective effects in SLE, but further research is needed to elucidate the precise mechanisms^17^.

The findings of this study suggest that medication use among patients with SLE, particularly the use of plaquenil (hydroxychloroquine), prednisolone, and mycophenolate mofetil, may have significant implications for cardiac function and biomarker levels. Hydroxychloroquine, a cornerstone of SLE treatment, has been shown to have cardioprotective effects, potentially contributing to the observed higher EF levels in patients with SLE. However, the immunosuppressive effects of prednisolone and mycophenolate mofetil, while essential for controlling disease activity, may also impact right ventricular function, as suggested by the lower TAPSE and pPTT levels in this study. The modulation of EPAS-1 and ghrelin levels by these medications is another area of interest as corticosteroids like prednisolone could influence these biomarkers, either directly or indirectly through their effects on inflammation and tissue oxygenation. This highlights the need for careful consideration of medication regimens in managing patients with SLE, particularly with regard to their potential impact on cardiovascular health. Further research is warranted to explore the long-term effects of these treatments on cardiac function and their interaction with biomarkers such as EPAS-1 and ghrelin.

The interplay between hormonal factors and SLE-related pathophysiology provides valuable context for interpreting our findings. Studies such as those by Lourenço et al. on adrenal steroidogenesis and ovarian reserve in childhood-onset patients with SLE, and by Soares-Jr et al. on the effects of hormone therapy in menopausal patients with SLE, underline the significant role of endocrine pathways in modulating disease activity and systemic inflammation in SLE^18,19^. These findings suggest that dysregulated steroidogenesis and hormonal fluctuations may contribute to the altered levels of EPAS-1 and ghrelin observed in our study, potentially influencing cardiac and vascular function in SLE. The higher levels of EPAS-1 in patients with SLE and increased disease activity or antiphospholipid positivity may reflect an adaptive response to hypoxic and pro-inflammatory states. Similarly, the cardioprotective properties of ghrelin might be modulated by hormonal interactions, underscoring the complex interdependencies between immune, endocrine, and cardiovascular systems in SLE.

In our study, the significantly lower pPTT values observed in patients with SLE compared with healthy controls highlight the potential utility of this parameter in assessing right ventricular dysfunction. This finding aligns with the study by Yavuz et al., which demonstrated the relationship between pulmonary arterial parameters and diastolic dysfunction in patients with heart failure with preserved ejection fraction, emphasizing the importance of noninvasive markers like pPTT in evaluating pulmonary hemodynamics and ventricular function^20^. The reduced pPTT in our cohort may reflect increased pulmonary vascular resistance or impaired pulmonary arterial compliance, both of which are associated with right ventricular strain. These results underscore the need for further investigation into pPTT as a marker of cardiovascular involvement in autoimmune diseases like SLE.

Overall, the findings of this study contribute to the growing body of evidence suggesting that both EPAS-1 and ghrelin play important roles in the cardiovascular complications of SLE. These biomarkers may offer valuable insights into the mechanisms underlying SLE-related right ventricular dysfunction and could potentially serve as targets for therapeutic intervention. Further large-scale studies are warranted to validate these findings and explore the potential of EPAS-1 and ghrelin as therapeutic targets in SLE.

### Limitations

This study has several limitations that should be acknowledged. First, the relatively small sample size may limit the generalizability of the findings to the broader population of patients with SLE. Second, the cross-sectional design of the study does not allow for the determination of causal relationships between elevated EPAS-1 and ghrelin levels and right ventricular dysfunction in SLE. Third, the study did not account for potential confounding factors such as variations in medication regimens or comorbid conditions, which could influence the levels of the biomarkers studied. Additionally, the reliance on echocardiographic assessments, while valuable, may not capture the full spectrum of cardiac involvement in SLE. An important limitation is that markers such as D-dimer and proBNP, which may be indicators of right ventricular functions, cannot be examined. The absence of cardiac MRI in the patient evaluations represents a limitation of the study. Additionally, the lack of follow-up data further limits the ability to assess long-term outcomes.

## CONCLUSION

This study underscores the complexity of cardiac involvement in SLE and highlights the need for a multifaceted approach to understanding and managing these complications. The identification of EPAS-1 and ghrelin as biomarkers of cardiac involvement offers a promising avenue for future research and underscores the importance of early detection and intervention in improving outcomes for patients with SLE.

## ETHICS APPROVAL AND INFORMED CONSENT

The study was reviewed and approved by the institutional research ethics board, adhering to the principles of the Helsinki Declaration. Written informed consent was obtained from all participants. Artificial intelligence-supported technologies were not used in the study.
